# 二维液相色谱-串联质谱法定量检测突发中毒生物样本中河豚毒素

**DOI:** 10.3724/SP.J.1123.2024.11026

**Published:** 2025-10-08

**Authors:** Li FANG, Fengmei QIU, Yuchao WANG

**Affiliations:** 1.舟山市疾病预防控制中心（舟山市卫生监督所），浙江 舟山 316021; 1. Zhoushan Municipal District Center for Disease Control and Prevention （Zhoushan Municipal Health Supervision Institute），Zhoushan 316021，China; 2.舟山市普陀区疾病预防控制中心，浙江 舟山 316100; 2. Putuo Center for Disease Control and Prevention，Zhoushan 316100，China

**Keywords:** 二维液相色谱, 质谱：血浆, 尿液, 生物样本, 河豚毒素, 中毒, two-dimensional liquid chromatography （2D-LC）, mass spectrometry （MS）, plasma, urine, biological samples, tetrodotoxin （TTX）, poisoning

## Abstract

河豚毒素是一种高毒性小分子神经毒素，可通过抑制神经细胞膜对Na^+^的通透性，影响神经信号传导，进而导致神经麻痹，严重者可因呼吸衰竭而死亡。我国部分沿海地区存在食用河豚鱼、织纹螺等海产品的习惯，因摄食含河豚毒素海产品导致中毒的事件时有发生。尽早识别毒素并进行对症解毒治疗可提高中毒患者的救治成功率，同时人体生物样本中毒素的浓度在一定程度上可以反映中毒程度和患者的预后状况。本研究建立了二维液相色谱-串联质谱法（2D-LC-MS/MS）定量检测突发中毒生物样本中河豚毒素的新方法。血浆或尿液样品经0.5%（v/v）乙酸乙腈溶液提取后高速离心，上清液先经第一维反相C_18_液相色谱柱粗分离，随后通过六通阀切换将目标物转移至亲水Amide液相色谱柱进行第二维色谱分离，电喷雾电离，选择反应监测（SRM）模式检测，基质匹配内标法定量。在0.2~40.0 μg/L（相当于生物样品中河豚毒素的含量为1.0~200.0 μg/L）范围内，河豚毒素呈现良好的线性关系，相关系数达到0.999 4以上；以3倍和10倍信噪比所对应的浓度为检出限和定量限时，血浆、尿液中河豚毒素的检出限均为0.3 μg/L，定量限均为1.0 μg/L；在2.0、10.0、50.0和200.0 μg/L的加标水平下，血浆和尿液中河豚毒素的日内回收率分别为84.4%~98.4%和84.4%~96.9%；日间回收率分别为87.7%~96.2%和84.8%~95.7%。检测方法日内、日间相对标准偏差均≤7.5%。该检测方法快速准确，无需复杂前处理，已成功应用于河豚毒素中毒生物样本的检测。

## Abstract (en)

Tetrodotoxin （TTX） is a powerful small-molecule neurotoxin primarily produced by specific marine endosymbiotic bacteria and can be enriched during symbiosis with aquatic organisms such as pufferfish， gastropods， and blue-ringed octopuses. TTX prevents sodium ions from entering nerve cells， which affects neuromuscular conduction and leads to progressive paralysis and even death due to respiratory failure. Poisoning ascribable to the ingestion of TTX-containing seafood has occurred occasionally in some coastal areas of China. The early identification of toxins and the administration of symptomatic detoxification therapies can improve the resuscitation success rates of poisoned patients. The concentration of TTX in clinical biological samples reflects the degree of patient poisoning and their prognosis. A method was established for the determination of the TTX in poisoned biological samples by two-dimensional liquid chromatography-tandem mass spectrometry （2D-LC-MS/MS）. A human plasma or urine sample （100 μL） was accurately pipetted into a 2-mL centrifuge tube， sequentially added a 10 mg/L kasugamycin solution （10 μL； internal standard）， ultrapure water （150 μL）， and 0.5% （v/v） acetic acid in acetonitrile （250 μL） as the extraction solvent， after which the mixture was subjected to vortex mixing at 2 200 r/min for 10 min and centrifugation for 10 min at 15 000 r/min and 4 ℃. The supernatant was roughly separated using a first-dimensional reverse-phase C_18_ column （Hypersil Gold C_18_， 50 mm×2.1 mm， 1.9 μm）. The target fraction was then transferred to a hydrophilic liquid chromatography column （Acquity UPLC BEH Amide， 150 mm×3.0 mm， 1.7 μm） via a six-way switching valve for second-dimensional separation and analysis using positive electrospray ionization and selected reaction monitoring （SRM） modes. Kasugamycin served as the internal standard for TTX quantitation， using matrix-matched calibration combined with the internal standard method. TTX exhibited good linearity in the 0.2-40.0 μg/L range （equivalent to 1.0-200.0 μg/L in biological samples）， with a correlation coefficient exceeding 0.999 4. The TTX in human plasma and urine samples exhibited matrix effects of 80.9% and 98.9%， respectively， with LODs and LOQs of 0.3 and 1.0 μg/L， respectively， determined for both sample types， based on three- and ten-times signal-to-noise ratios， respectively. The TTX in human plasma and urine exhibited intra-day recoveries of 84.4%–98.4% and 84.4%–96.9%， respectively， with inter-day recoveries of 87.7%–96.2% and 84.8%–95.7%， respectively， at spiked levels of 2.0， 10.0， 50.0， and 200.0 μg/L. Intra-day relative standard deviations （RSDs） of 3.2%–7.2% and 2.9%–5.7% were recorded for TTX in human plasma and urine， respectively， with inter-day RSDs of 2.3%–3.2% and 1.0%–7.5%， respectively. The intra-day and inter-day RSDs of both sample types were determined to be lower than 7.5%. The method is accurate， fast， avoids complicated pretreatment steps， and was successfully used to detect TTX in food-poisoning scenarios.

河豚毒素（tetrodotoxin，TTX）是一种高毒性小分子神经毒素，主要由某些海洋内生细菌产生，可在河豚鱼、织纹螺、鲎、蓝环章鱼等海洋生物体内富集^［1］^。河豚毒素主要通过抑制神经细胞膜对Na^+^的通透性，阻止Na^+^进入神经细胞，影响神经信号传导，进而导致神经麻痹，严重者可因呼吸衰竭而死亡^［2］^。我国部分沿海地区存在食用河豚鱼、织纹螺等海产品的习惯，因摄食含河豚毒素海产品导致中毒的事件时有发生^［3］^，其主要临床症状为震颤、口舌麻木、四肢无力和瘫痪等，目前尚无特效药可用于解毒治疗，只能进行催吐洗胃及对症支持治疗^［4］^。尽早识别毒素并进行对症解毒治疗可提高中毒患者的救治成功率，同时人体生物样本中毒素的浓度在一定程度上可以反映中毒程度和患者的预后状况^［5］^。在突发食物中毒事件调查取样过程中，时常无法获得中毒食物样本，中毒病人血液、尿液等生物样本是较易获得且可用于检测的样本。因此，开发和建立一种灵敏、准确定量检测人体生物样本中河豚毒素的方法显得十分必要。

目前，关于人体生物样本中河豚毒素的检测方法主要有免疫层析法^［6］^、电化学传感器法^［7，8］^、高效液相色谱-紫外检测法（HPLC-UV）^［9］^、高效液相色谱-荧光检测法（HPLC-FLD）^［10］^、气相色谱-质谱法（GC-MS）^［11］^和液相色谱-串联质谱法（LC-MS/MS）^［5，12-19］^。其中，免疫层析法主要用于现场快速检测，非确证方法；电化学传感器法所需的酶和适配体易受环境条件影响而失活，进而导致传感器的重现性和稳定性降低；HPLC-UV灵敏度相对较低，在样品基质复杂的情况下易受基质干扰；HPLC-FLD和GC-MS均需要衍生化处理，测试条件相对复杂；LC-MS/MS是一种常用的定量检测方法，具有灵敏度高、选择性好、无需衍生化等优势。

当前，人体生物样本中河豚毒素的主要前处理方法有直接蛋白质沉淀法^［12］^、分散固相萃取法^［13］^、固相萃取法^［5，14-19］^等。常用的固相萃取柱有C_18_柱^［14］^、HILIC柱^［15，16］^、甲基丙烯酸酯-苯乙烯二乙烯基苯柱^［17］^、羧酸乙基柱串联酰胺柱^［18］^、混合阳离子交换柱^［19］^、免疫亲和柱^［5］^等。尽管上述前处理方法净化效果较好，但存在溶剂消耗量大、成本高、操作要求高、劳动强度大、前处理时间长等缺陷。二维液相色谱（2D-LC）是一种将分离机制不同而又相互独立的两根色谱柱串联构成的分离系统，具有峰容量大、能降低复杂样品基质效应、可实现样品自动化分析等优点，已成功应用于尿液中鱼藤酮、8-羟基脱氧鸟苷的检测^［20，21］^。

本研究利用二维液相色谱分离，结合阀切换技术，三重四极杆质谱检测，实现了快速准确定量检测血浆、尿液中河豚毒素的目的。

## 1 实验部分

### 1.1 仪器与试剂

TSQ Vantage三重四极杆质谱仪，配有电喷雾离子源（ESI源，美国Thermo公司）；Transcend TLX-1系统（美国Thermo公司）由 PAL自动进样系统（瑞士CTC公司）、2套Dionex U3000型液相色谱泵组成；Microfuge 20R型小容量高速冷冻离心机（美国Beckman公司）；DMT-2500型涡旋混合器（杭州米欧公司）；Milli-Q超纯水（18.2 MΩ·cm）处理系统（德国Merck公司）。

甲酸、乙酸和甲酸铵均为LC-MS级，购自德国CNW公司；甲醇、乙腈均为HPLC级，购自德国Merck公司。河豚毒素（100 mg/L，上海安谱公司）；春雷霉素（kasugamycin，纯度98.9%，北京振翔公司）；伏格列波糖（voglibose，10 mmol/L）和L-精氨酸-^15^N_4_（L-arginine-^15^N_4_，纯度≥98.5%）购自上海阿拉丁生化科技股份有限公司。空白尿样来自健康志愿者，空白血浆样品购自美国Innovative Research公司。中毒病人血浆和尿液样本各1份，于2022年7月26日由舟山医院提供，样本于-20 ℃冷冻保存。本研究进行的所有程序均符合伦理学要求，并经舟山市疾病预防控制中心伦理审查委员会批准，批准编号为Zs24-03。

### 1.2 样品制备

准确吸取100 μL尿液或血浆样品（冷冻保存样品需恢复至室温后取用），置于2 mL离心管中，接着依次加入10 μL内标溶液（春雷霉素：10 mg/L）、150 μL超纯水和250 μL 0.5%乙酸乙腈溶液，涡旋振荡提取10 min，在4 ℃下以15 000 r/min的转速离心10 min，吸取上清液转移至样品瓶待测。

### 1.3 空白基质制备

吸取与待测样品基质相同，不含河豚毒素的试样于离心管中。按照1.2节中步骤进行提取，制备获得空白基质溶液。

### 1.4 标准溶液的配制

河豚毒素标准溶液用0.1%甲酸水溶液稀释，配成质量浓度为1.0 μg/mL的标准储备液。春雷霉素内标溶液用50%乙腈水溶液稀释。用空白基质配制成质量浓度为0.2、0.4、1.0、2.0、4.0、10.0、20.0和40.0 μg/L（相当于血浆、尿液中含量为1.0、2.0、5.0、10.0、20.0、50.0、100.0和200.0 μg/L）的系列基质匹配溶液。

### 1.5 二维液相色谱分离条件

第一维液相色谱：Hypersil GOLD C_18_色谱柱（50 mm×2.1 mm， 1.9 μm，美国Thermo公司），上样泵的流动相A为0.1%甲酸水溶液，流动相 B 为乙腈，进样量为20 μL。梯度洗脱程序见表1（Pump 1），二维液相色谱管路连接图如图1。

**表1:** 二维液相色谱梯度洗脱程序

Step No.	Time/min	Function	Pump 1			Pump 2		
			Flow rate/ （mL/min）	*φ*（A）/%	*φ*（B）/%	Flow rate/ （mL/min）	*φ*（C）/%	*φ*（D）/%
1	0	loading	0.1	98	2	0.3	5	95
2	1.17	transferring	0.1	98	2	0.4	5	95
3	2.40	washing	0.2	10	90	0.3	5	95
4	3.40	equilibrating	0.2	98	2	0.3	5	95
5	13.4	equilibrating	0.3	98	2	0.3	60	40
6	14.4	equilibrating	0.1	98	2	0.3	5	95
7	15.5	equilibrating	0.1	98	2	0.3	5	95

A： 0.1% formic acid aqueous solution； B： acetonitrile； C： water with 0.1% formic acid and 2 mmol/L ammonium formate； D： 2 mmol/L ammonium formate containing 0.1% formic acid-acetonitrile （5∶95， v/v）.

**图1:**
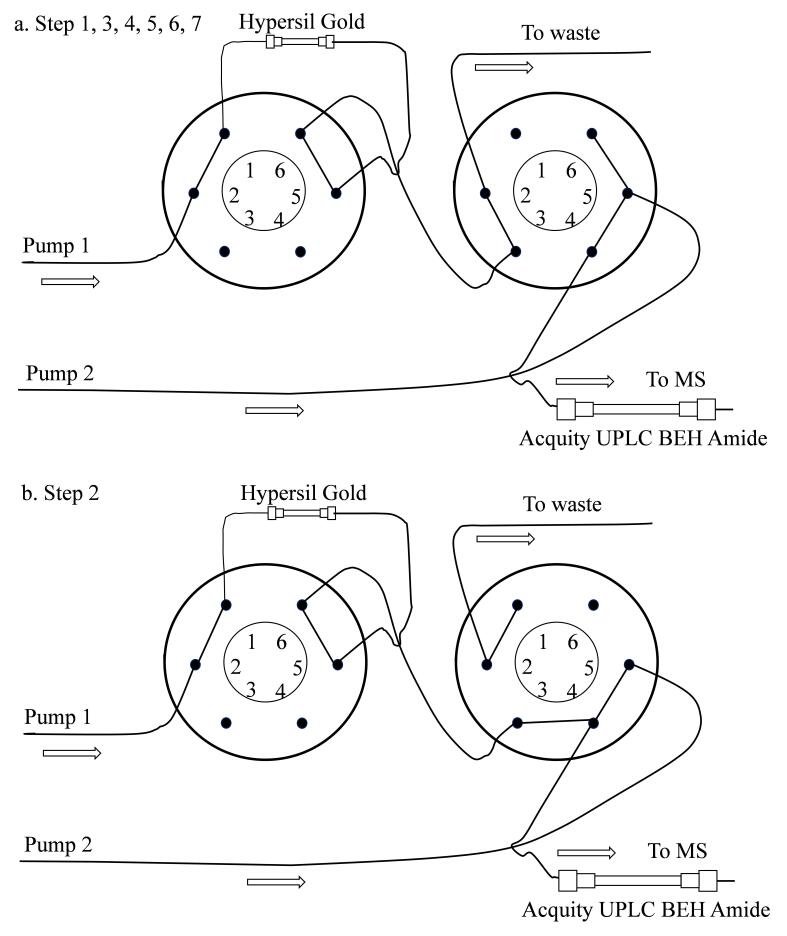
二维液相色谱管路连接示意图

第二维液相色谱：Acquity UPLC BEH Amide色谱柱（150 mm × 3.0 mm， 1.7 μm，美国Waters公司），流动相C为含0.1%甲酸的2 mmol/L甲酸铵水溶液，D为含0.1%甲酸的2 mmol/L甲酸铵水溶液-乙腈（5∶95， v/v），梯度洗脱程序见表1（Pump 2）。

### 1.6 质谱条件

质谱采用电喷雾离子源（ESI），正离子扫描方式，选择反应监测（SRM）模式；喷雾电压为3.0 kV；汽化温度为300 ℃；离子传输毛细管温度为325 ℃；鞘气压为45 arb，辅助气压为15 arb，这两种雾化气均为高纯氮气；碰撞气为高纯氩气，压力为0.2 Pa（1.5 mTorr）。使用前调节各气体流量使质谱灵敏度达到检测要求。河豚毒素质谱检测参数如下：定量离子对为320.0>302.0，碰撞能量24 eV；定性离子对为320.0>162.0，碰撞能量37 eV；透镜（S-lens）电压为108 V。内标春雷霉素的离子对为380.1>112.1，碰撞能量17 eV；S-lens电压为78 V。

## 2 结果与讨论

### 2.1 二维液相色谱条件优化

二维液相色谱由2套液相色谱系统组成，第一维液相色谱用于样品的粗分离，第二维液相色谱用于样品的分析检测。河豚毒素是一种小分子极性化合物，反相C_18_柱对目标物的保留相对较弱，本文选用C_18_柱在低有机相条件下对样品进行粗分离，将目标物色谱峰通过六通阀切割转移至第二维液相色谱，将对C_18_柱有保留的杂质保留在色谱柱中（图2）。参照本课题组先前研究结果^［13，22］^，选择对目标物有较强保留的亲水作用色谱柱（Acquity UPLC BEH Amide）用于第二维液相色谱分离。

**图2:**
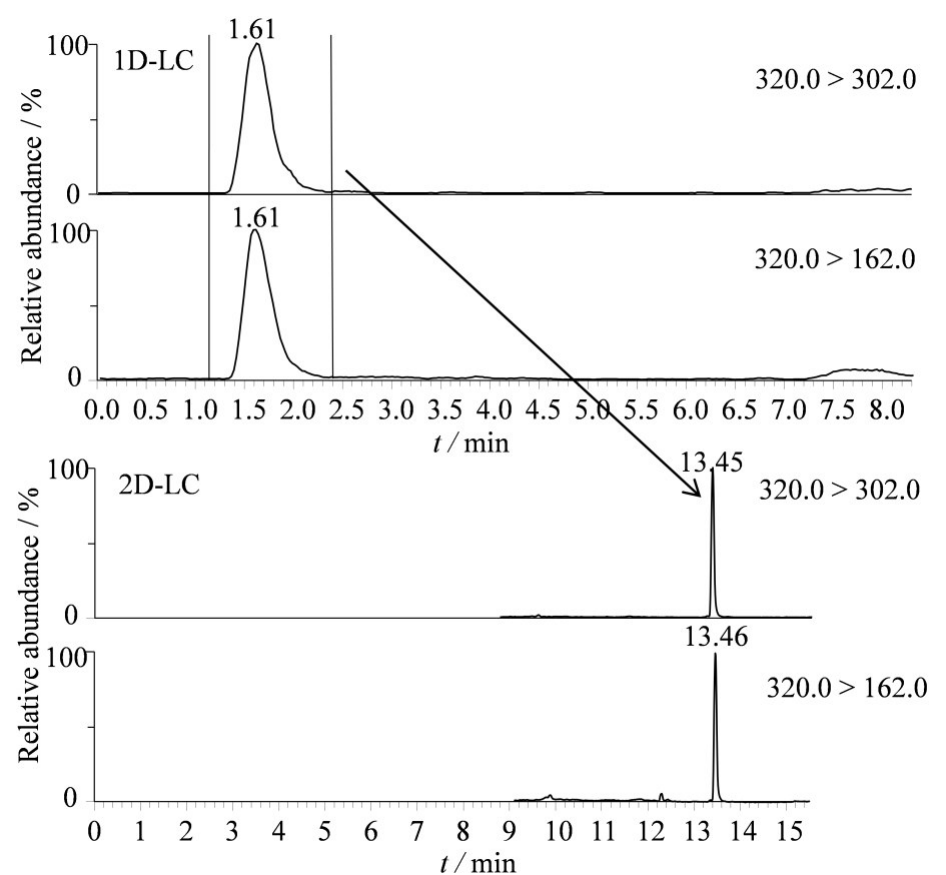
河豚毒素的二维液相色谱图

由于二维液相色谱所使用的2根色谱柱填料性质上存在明显差异，第一维液相色谱流出液转移至第二维液相色谱柱时的流动相组成会对目标物色谱峰形产生影响。本研究第一维液相色谱采用0.1%甲酸水溶液-乙腈（98∶2，v/v），以0.1 mL/min的流速分离并携带目标物转移至第二维液相色谱，高比例水相的流动相组成直接进入第二维液相色谱柱，不利于目标物在该色谱柱里的保留，需要通过改变第二维液相色谱流动相（高有机相比例）的流速达到提高目标物转移时流动相中有机相比例的目的。本文对比了目标物转移过程中（Step 2）第二维液相色谱（Pump 2）流速（0.2、0.3、0.4、0.5和0.6 mL/min）对目标物色谱保留行为的影响。从图3可以看出，当第二维液相色谱的流速为0.2 mL/min和0.3 mL/min时目标物色谱峰出现严重前倾，这是由于低有机相比例导致目标物未能在亲水色谱柱上实现较好保留；当流速为0.5 mL/min和0.6 mL/min时，也出现色谱峰前倾的现象，这可能是由于色谱柱流速过高导致小部分目标物未实现较好保留提前从色谱柱流出；当流速为0.4 mL/min时，目标物的色谱峰峰形尖锐、对称。因此本文将0.4 mL/min设定为目标物转移时第二维液相色谱的流速。

**图3:**
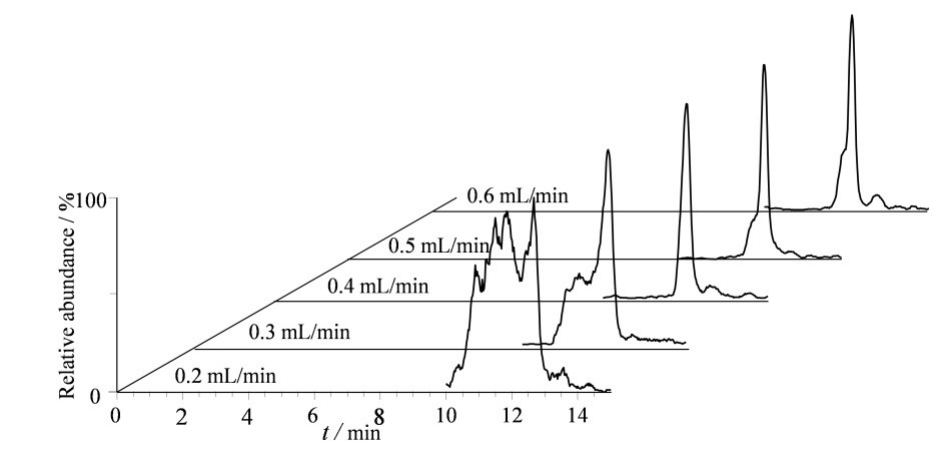
第一维液相色谱转移至第二维液相色谱时第二维液相色谱流动相的流速对河豚毒素色谱峰峰形的影响

### 2.2 内标物的选择

目标物对应的同位素内标是样品分析检测最理想的内标，但当前河豚毒素的同位素内标尚未在市场上销售。参考已发表文献，一些结构相似的化合物，如11-脱氧河豚毒素^［12］^、L-精氨酸-^15^N_4_^［16］^、春雷霉素^［19］^、伏格列波糖^［23］^等，已被选为河豚毒素的内标。尽管11-脱氧河豚毒素与河豚毒素的结构最为相似，但未在市场供应。本研究对比了伏格列波糖、L-精氨酸-^15^N_4_和春雷霉素这3种内标物。从图4可知，春雷霉素在二维液相色谱中的色谱行为和保留时间与目标物相近，且色谱峰峰形最优。因此，春雷霉素被选为生物样本中河豚毒素检测的内标物。

**图4:**
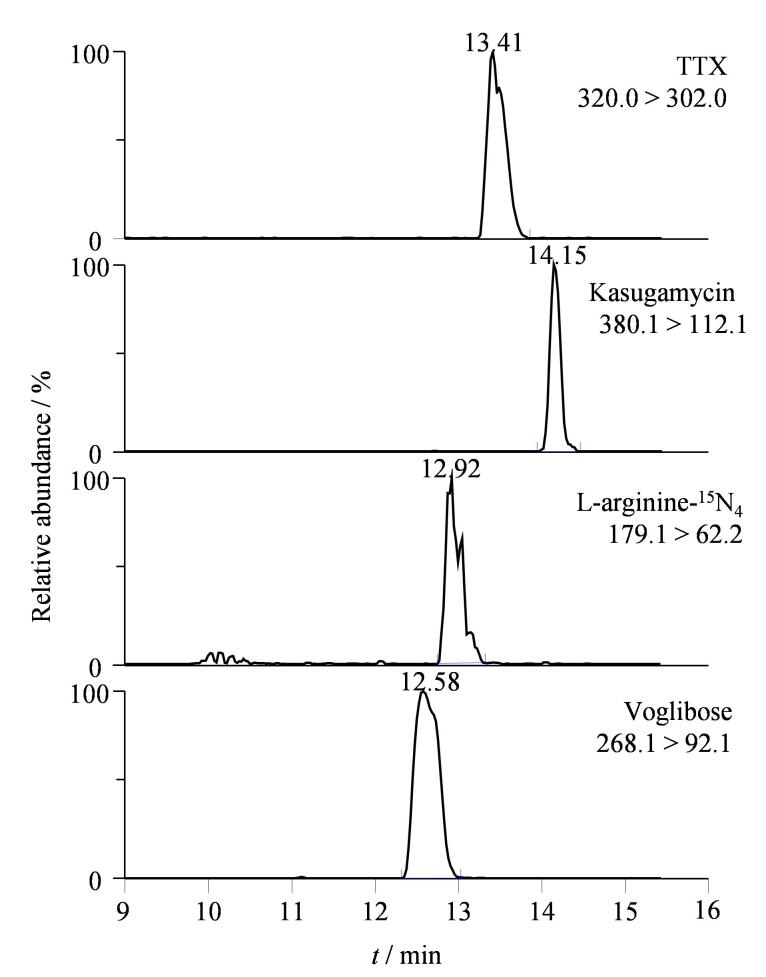
河豚毒素及其3种内标化合物的色谱图

### 2.3 提取条件的优化

血浆、尿液等生物样品基质中含有大量的蛋白质和无机盐，乙腈是生物基质中蛋白质和盐类沉淀最有效的溶剂之一，但对于盐类含量较高的尿液而言，单纯使用乙腈提取时，试管底部会出现微量的水层，导致水溶性的目标物提取效率降低，需加入适量的纯水来降低样品中盐的浓度，以促进提取溶剂与样品的互溶^［19］^。对于蛋白质含量较高的血浆样品而言，直接用乙腈提取时易出现抱团沉淀的现象，导致部分目标物在振荡提取过程中无法充分释放，血浆样品提取前加入适量的纯水能有效降低抱团沉淀现象的出现。本研究对比了乙腈及酸化乙腈（0.1%甲酸乙腈、0.5%甲酸乙腈、0.1%乙酸乙腈和0.5%乙酸乙腈）作为提取溶剂对血浆和尿液中目标毒素提取回收率的影响。从图5可知，无论对于血浆样品还是尿液样品，用乙腈提取时目标毒素的提取回收率均低于80%。当在乙腈提取溶剂中加入甲酸或乙酸后，目标毒素的提取回收率呈现明显提高的现象。这是由于河豚毒素具有弱碱性，在酸性条件下带正电，易被酸性溶剂提取。当0.5%乙酸乙腈为提取溶剂时，血浆、尿液基质中目标物均可获得较高的提取回收率。因此，本研究选择0.5%乙酸乙腈作为生物样本中河豚毒素的提取溶剂。

**图5:**
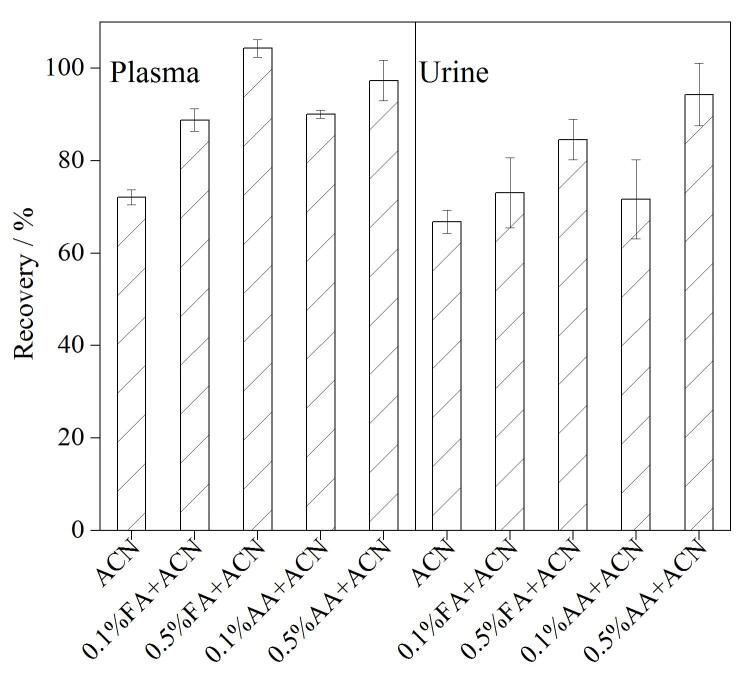
提取溶剂对河豚毒素回收率的影响（*n*=3） FA： formic acid； AA： acetic acid； ACN： acetonitrile.

### 2.4 基质效应、工作曲线及检出限

按1.4节方法配制基质匹配溶液并测定，以河豚毒素的浓度为横坐标，目标物峰面积与内标物峰面积的比值为纵坐标，绘制基质工作曲线，在0.2~40.0 μg/L（相当于生物样品中河豚毒素的含量为1.0~200.0 μg/L）范围内线性相关性良好，相关系数（*R* ^2^）大于0.999 4。本研究采用基质匹配工作曲线斜率与纯溶剂标准曲线斜率的百分比值来评价检测方法的基质效应（ME）。结果显示，血浆和尿液基质的ME值分别为80.9%和98.9%，表明血浆基质存在弱基质抑制效应。本文采用基质匹配内标法定量测定实际样品，可补偿基质效应对样品定量检测的影响。方法的检出限和定量限分别基于3倍和10倍信噪比（*S/N*）获得，血浆、尿液基质中河豚毒素的检出限均为0.3 μg/L，定量限均为1.0 μg/L。

### 2.5 回收率和精密度

本研究以血浆和尿液为研究对象，考察检测方法的回收率和精密度。准确称取空白血浆和尿液样品各100 μL，设定4个添加水平（2.0、10.0、50.0和200.0 μg/L），根据上述方法进行检测，每组样品6个平行，测定日内回收率和日内相对标准偏差（RSD）；每天测试1批次，连续3天，共检测3批次，测得日间回收率和日间精密度。由表2可知，血浆和尿液中河豚毒素的日内回收率分别为84.4%~98.4%和84.4%~96.9%；日间回收率分别为87.7%~96.2%和84.8%~95.7%，检测方法的日内和日间RSD分别≤7.2%和7.5%。

**表2:** 血浆和尿液中河豚毒素的加标回收率和精密度

Toxin	Spiked/（μg/L）	Plasma				Urine			
		Intra-day （*n*=6）		Inter-day （*n*=3）		Intra-day （*n*=6）		Inter-day （*n*=3）	
		Recovery/%	RSD/%	Recovery/%	RSD/%	Recovery/%	RSD /%	Recovery/%	RSD/%
TTX	2.0	92.1	6.1	91.5	2.7	84.4	5.7	84.8	3.8
	10.0	89.4	7.2	91.2	2.3	91.1	3.0	89.8	7.5
	50.0	98.4	6.0	96.2	2.6	93.9	2.9	93.7	1.3
	200.0	84.4	3.2	87.7	3.2	96.9	5.2	95.7	1.0

### 2.6 与已发表检测方法比较

表3列出了已发表的液相色谱-质谱检测生物样品中河豚毒素的方法。与这些方法比较，本研究检测方法的取样量相对较少（100 μL），但方法检出限或定量限能达到与已报道方法相当的水平。此外，Tsai等^［14］^报道中毒病人尿液和血中河豚毒素的含量分别为15~110 μg/L和1.4~13 μg/L；徐小民等^［19］^检测了河豚鱼和织纹螺中毒患者尿样11份，河豚毒素的含量范围为 0.4~138 μg/L；张秀尧等^［5］^对河豚毒素中毒患者的8份尿液样品进行重新测定，含量为1.0~148 μg/L。与上述中毒样品中河豚毒素的含量比较，本方法的检测灵敏度和线性范围均能满足中毒检测需求，具有良好的实际应用价值。

**表3:** 与已发表检测方法的比较

Ref.	Matrix	Sample volume/μL	Clean-up method	Analytical method	LC runtime/min	Linear range/（μg/L）	LOD/（μg/L）	LOQ/（μg/L）
［^5^］	urine	1000	SPE with immunoaffinity column	LC-MS/MS	6.5	0.05-400	0.02	0.05
	plasma					0.05-400	0.02	0.05
［^12^］	urine	500	protein precipitation	LC-MS/MS	16	0.986-98.6	NF	0.986
［^14^］	blood	1000	SPE with C_18_ cartridge	LC-MS	10	30-3000	5	NF
	urine					30-3000	5	NF
［^15^］	serum	1000	SPE with C_18_ and HILIC cartridge	LC-MS/MS	6.5	10-500	0.13	2.5
	urine					2.5-20	0.13	2.5
［^16^］	plasma	100	SPE with Siphila i HILIX 96 well plate	LC-MS/MS	4.5	0.1-20	NF	0.1
［^17^］	serum	1000	SPE with methacrylate styrenedivinyl benzene cartridge	LC-MS/MS	10	0.1-20	0.1	NF
	urine					0.1-20	0.1	NF
［^18^］	serum	100	SPE with monospin CBA cartridge	LC-MS/MS	4	1-25	0.5	1
	urine		SPE with monospin amide cartridge			0.5-200	0.25	0.5
［^19^］	urine	400	SPE with MCX cartridge	LC-MS/MS	12	0.2-200	0.1	0.2
This study	plasma	100	protein precipitation	2D-LC-MS/MS	15.5	1.0-200.0	0.3	1.0
	urine					1.0-200.0	0.3	1.0

NF： not found.

### 2.7 实际中毒样品检测

应用本方法对本市一起因误食织纹螺导致的突发食物中毒事件中涉及病人的生物样本进行了分析检测。中毒病人血浆和尿液中均检出河豚毒素，含量分别为2.1 μg/L和123 μg/L，中毒生物样本色谱图见图6。该病人送医后一度处于昏迷状态，由于中毒原因识别及时，经过一段时间的对症脱毒治疗后，该中毒病人最终康复出院。通过实际中毒样品检测，再次验证了方法的有效性。河豚毒素作为一种具有强烈的急性毒性的生物毒素，摄入后潜伏期一般为0.5~2 h^［24］^。在河豚毒素中毒事件中，尿液和血浆都是重要的生物样本基质。尿液是河豚毒素在人体内排泄的主要途径，在中毒患者尿液样本通常能检测到较高浓度的河豚毒素，适合在中毒事件中判定中毒原因，但尿液中河豚毒素的浓度与患者的中毒程度无明显关系^［24］^。就血液而言，中毒患者血中的河豚毒素浓度虽然显著低于尿液，但有以往的文献报道显示血中的河豚毒素浓度与麻痹作用、呼吸停止的发生存在一定关系^［24，25］^。Islam等^［25］^研究认为当血液中河豚毒素含量超过9 μg/L就可能致命。对于实际中毒事件，尽早采集生物样本并开展相关毒素检测确定中毒原因，可为中毒患者制定更具针对性的治疗方案，提高救治成功率。

**图6:**
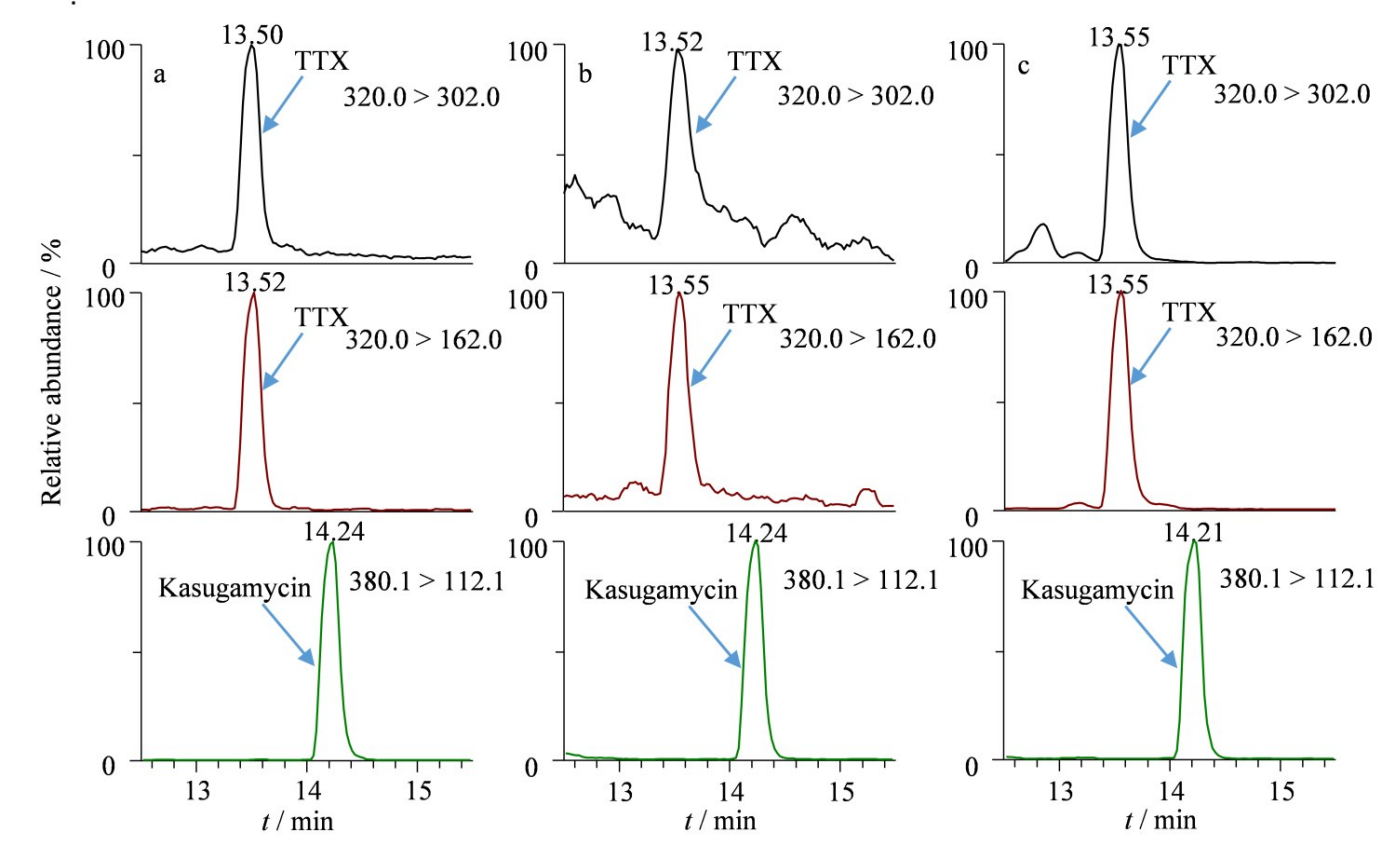
河豚毒素的色谱图 a. standard solution at 2 μg/L； b. plasma from poisoned patient； c. urine from poisoned patient.

## 3 结论

本方法采用0.5%乙酸乙腈溶液提取后直接进样，二维液相色谱分离，三重四极杆质谱检测，内标法定量，实现中毒病人血浆、尿液中河豚毒素的定量测定。方法学验证结果表明，本法灵敏度高，重复性好，定量准确性高，应用于实际样品检测取得了满意的结果，适用于河豚毒素中毒应急检测和临床监测。
